# Heterocyclic Compounds as Dipeptidyl Peptidase-IV Inhibitors with Special Emphasis on Oxadiazoles as Potent Anti-Diabetic Agents

**DOI:** 10.3390/molecules27186001

**Published:** 2022-09-15

**Authors:** Badrud Duza Mohammad, Mirza Shahed Baig, Neeraj Bhandari, Falak A. Siddiqui, Sharuk L. Khan, Zubair Ahmad, Farhat S. Khan, Priti Tagde, Philippe Jeandet

**Affiliations:** 1 Department of Pharmaceutical Chemistry, G R T Institute of Pharmaceutical Education and Research, GRT Mahalakshmi Nagar, Tiruttani 631209, Tamil Nadu, India; 2 Department of Pharmaceutical Chemistry, Y. B. Chavan College of Pharmacy, Aurangabad 431001, Maharashtra, India; 3 Arni School of Pharmacy, Arni University, Kathgarh, Indora 176401, Himachal Pradesh, India; 4 Department of Pharmaceutical Chemistry, N.B.S. Institute of Pharmacy, Ausa 413520, Maharashtra, India; 5 Unit of Bee Research and Honey Production, Faculty of Science, King Khalid University, P.O. Box 9004, Abha 61413, Saudi Arabia; 6 Biology Department, College of Arts and Sciences, Dehran Al-Junub, King Khalid University, P.O. Box 9004, Abha 61413, Saudi Arabia; 7 Patel College of Pharmacy, Madhyanchal Professional University, Bhopal 462044, Madhya Pradesh, India; 8 Research Unit Induced Resistance and Plant Bioprotection, University of Reims, USC INRAe 1488, SFR Condorcet FR CNRS 3417, 51687 Reims, France

**Keywords:** oxadiazole, sulfonylureas, DPP-IV inhibitors, diabetes mellitus, peptidomimetics

## Abstract

Dipeptidyl peptidase-IV (DPP-IV) inhibitors, often known as gliptins, have been used to treat type 2 diabetes mellitus (T2DM). They may be combined with other medications as an additional treatment or used alone as a monotherapy. In addition to insulin, sulfonylureas, thiazolidinediones, and metformin, these molecules appear as possible therapeutic options. Oxadiazole rings have been employed in numerous different ways during drug development efforts. It has been shown that including them in the pharmacophore increases the amount of ligand that may be bound. The exceptional hydrogen bond acceptor properties of oxadiazoles and the distinct hydrocarbon bonding potential of their regioisomers have been established. Beside their anti-diabetic effects, oxadiazoles display a wide range of pharmacological properties. In this study, we made the assumption that molecules containing oxadiazole rings may afford a different approach to the treatment of diabetes, not only for controlling glycemic levels but also for preventing atherosclerosis progression and other complications associated with diabetes. It was observed that oxadiazole fusion with benzothiazole, 5-(2,5,2-trifluoroethoxy) phenyl, β-homophenylalanine, 2-methyl-2-{5-(4-chlorophenyl), diamine-bridged *bis*-coumarinyl, 5-aryl-2-(6′-nitrobenzofuran-2′-yl), nitrobenzofuran, and/or oxindole leads to potential anti-diabetic activity.

## 1. Introduction

Dipeptidyl peptidase-IV (DPP-IV) inhibitors, often known as gliptins, are a class of oral anti-diabetic medications that have been given approval by the Food and Drug Administration (FDA) to treat individuals with type 2 diabetes mellitus (T2DM) [1]. Following oral ingestion of food, the production of incretin hormones, which are hormones that are vital for maintaining glucose homeostasis, occurs in the gut. These hormones are the targets through which these drugs act [2]. Independent of the incretin pathway, in addition to their antihyperglycemic activities, this family of drugs also displays antihypertensive properties [3], anti-inflammatory effects [4], and antiapoptotic effects [5], as well as immunomodulatory effects [6,7] on the heart, kidneys, and blood vessels. A number of studies have demonstrated that this class of molecules may also be useful for treating transplant patients who have acquired new-onset diabetes after their transplantation (NODAT). These molecules may be combined with other medications as an additional treatment or used alone as a monotherapy. Sulfonylureas, thiazolidinediones, and metformin are also possible therapeutic options in addition to insulin [8,9]. Iminosugars represent a kind of polyhydroxylated secondary or tertiary amines that mimic monosaccharide sugars, but which contain nitrogen in lieu of oxygen in the ring. Iminosugars constitute a wide variety of different types of small organic molecules. These molecules belong to the class of pyrrolidines, piperidines, azepanes, nortropanes, pyrrolizidines, and indolizidines. The capacity of iminosugars to function as inhibitors of glycosidases and glycosyltransferases constituted the first role they were utilized in. These inhibitors work mostly through competitive inhibition and are mainly used to treat diabetes [10,11,12,13,14].

**Figure 1:**
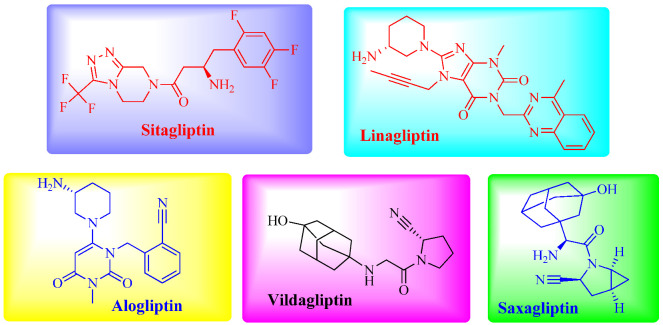
Structures of clinically-approved DPP-IV inhibitors.

The glucagon-like peptide-1 (GLP-1) has recently emerged as a possible target for T2DM treatment. GLP-1 is produced by the gut after a meal, enhancing insulin output. It has been demonstrated that increased levels of GLP-1 may contribute to a better glycemic control in T2DM individuals. DPP-IV controls the activity of GLP-1 by cleaving the *N*-terminus of GLP-1 [7,8,9,10,11,12,13,14,15,16,17,18,19,20,21,22,23,24,25,26,27,28,29,30,31,32,33,34,35,36]-amide to generate inert GLP-1 [9,10,11,12,13,14,15,16,17,18,19,20,21,22,23,24,25,26,27,28,29,30,31,32,33,34,35,36]-amide. The quantity of GLP-1 in the circulation may be increased by inhibiting DPP-IV [15]. As a result, a significant amount of time and effort has been dedicated to the research of DPP-IV inhibitors as potential treatments of T2DM. Sitagliptin [16] is one of these drugs, and it has been available in the United States since 2006 as the first FDA-approved DPP-IV inhibitor. Saxagliptin [17,18], vildagliptin [19], alogliptin [20], and linagliptin [21] (Figure 1) are all medications that have either been studied in diabetic patients or approved for sale in some countries. Based on structural similarities or differences with the DPP-IV molecule, DPP-IV inhibitors may be classified as either peptidomimetics (vildagliptin and saxagliptin) or non-peptidomimetics (sitagliptin, alogliptin, linagliptin). They are reversible competitive inhibitors of the DPP-IV substrates. These compounds have various degrees of affinity with DPP-IV substrates. The selectivity of the peptidomimetics toward DPP-IV is often lower as compared to DPP8/9. The greater the relative inhibition of DPP8/9 and the lower the relative selectivity toward DPP-IV, the greater the risk of adverse consequences (allergic skin manifestations, etc.) [22,23]. When compared to non-peptidomimetics, peptidomimetics display a distinct DPP-IV inhibition mechanism. Because non-peptidomimetics generate non-covalent extra-cellular interactions with residues in the catalytic region of the DPP-IV substrate, their inhibiting activity is powerful and rapid. In contrast, inhibition of the DPP-IV substrate by peptidomimetics takes place in a mode that includes the development of a reversible covalent enzyme-inhibitor complex. This complex may be broken down again. This complex binds to and dissociates from the catalytic site of the DPP-IV substrate in a very slow manner, resulting in the persistent inhibition of the DPP-IV enzyme a long time after the medication has been rendered ineffective. Because of this, the catalytic activity is hindered long after the free drug has been eliminated from circulation. In fact, vildagliptin and saxagliptin may be able to suppress DPP-IV activity for a longer period while having relatively short half-lives [24,25]. 

The usage of gliptin has been proven to be equally effective as compared to metformin, sulfonylureas (glimepiride, glipizide), thiazolidinediones (rosiglitazone, pioglitazone), and alfa-glucosidase inhibitors (voglibose). Compared to sulfonylureas, this therapy almost never causes hypoglycemia or affects body weight [26,27,28]. According to the findings of a meta-analysis that compared the effectiveness of sitagliptin and vildagliptin, the reported total HbA1c decrease was ~0.74% and 0.73%, respectively. If the starting HbA1c was superior to 9% instead of 8%, the glycemic results were shown to be more favorable [22]. According to a recent meta-analysis, the use of a gliptin was associated with a larger percentage of patients reaching the HbA1c target of <7% without any weight gain or hypoglycemia [29]. We have thus reviewed here the possible utilization of heterocyclic compounds as DPP-IV inhibitors, with special emphasis on oxadiazoles as potent anti-diabetic agents.

## 2. Review of Different Heterocyclic Compounds as Potent DPP-IV Inhibitors

**Figure 2:**
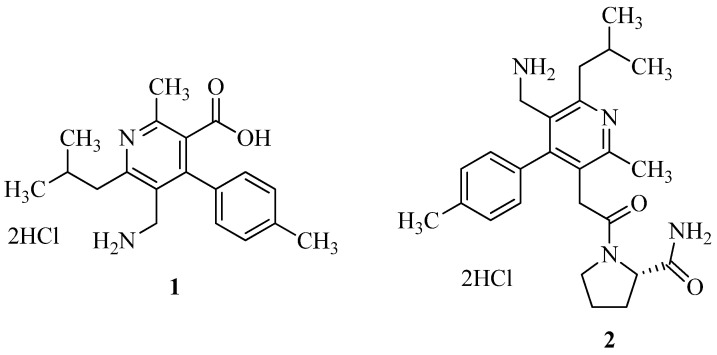
3-Pyridylacetamide derivatives as DPP-IV inhibitors.

Yasufumi Miyamoto et al. [15] used structure-based drug design (SBDD) to modify compound **1** to generate a novel and strong DPP-IV inhibitor for the treatment of T2DM. Using the results of X-ray co-crystallography studies on compound 1 revealed that Arg125 might be a suitable target amino acid residue for obtaining a stronger inhibitory activity. According to the theory, the guanidino group of Arg125 may engage bidentately with two separate hydrogen bond acceptors at the same time. As a result, scientists have synthesized a number of 3-pyridylacetamide derivatives that each include an extra hydrogen bond acceptor and have the potential to take part in bidentate interactions with Arg125. Compound **2** was shown to bind in a bivalent manner with the guanidino group of Arg125, which is a powerful and selective DPP-IV inhibitor. The structures of compounds **1** and **2** are illustrated in Figure 2.

**Figure 3:**
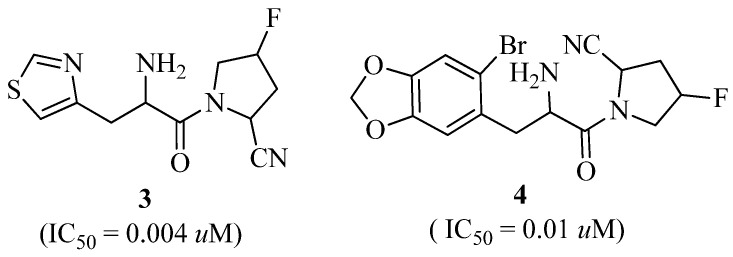
Pyrrole-2-carbonitrile has been shown to be effective as a DPP-IV inhibitor.

Utilizing the SAR of pyrrole-2-carbonitrile inhibitors, a variety of new hetero-aromatic moiety-substituted amino pyrrole-2-carbonitrile derivatives were synthesized. IC_50_ values ranged from 0.004 to 113.6 μM for all drugs, indicating that they were effective DPP-IV inhibitors. Compounds 3 (IC_50_ = 0.004 μM) and 4 (IC_50_ = 0.01 μM) were found to have excellent inhibitory activities against DPP-IV and a good efficacy in an oral glucose tolerance test (OGTT) in mice. Moreover, compounds **3** and **4** exhibited intermediate pharmacokinetic characteristics (**3**, F% = 37.8%, t_1/2_ = 1.45 h; **4**, F% = 16.8%, t_1/2_ = 3.64 h). The structures of compounds **3** and **4** are displayed in Figure 3 [30].

**Figure 4:**
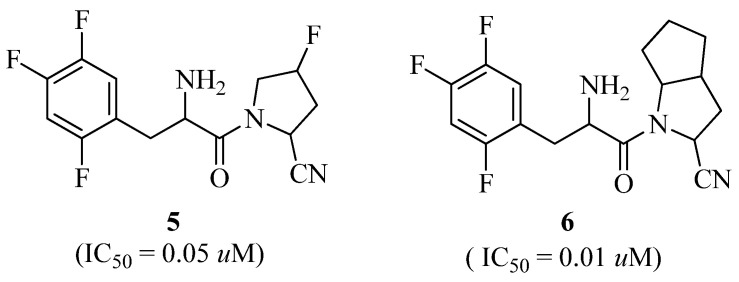
β-amino pyrrole-2-carbonitrile analogues.

New beta-amino pyrrole-2-carbonitrile derivatives have been discovered and developed using the rational drug design approach. Compounds **5** and **6** (Figure 4) were reported to be powerful and specific DPP-IV inhibitors in vivo, leading to a reduction in glucose amounts in the blood. Compound **5** significantly inhibited DPP-IV (IC_50_ = 0.05 μM), and also displayed a high oral bioavailability (F = 53.2%). Compound **6** demonstrated strong DPP-IV inhibitory action (IC_50_ = 0.01 μM), high selectivity against related peptidases, good effectiveness during the oral glucose tolerance test (OGTT) in ICR mice, and a moderate pharmacokinetic profile [31].

**Figure 5:**
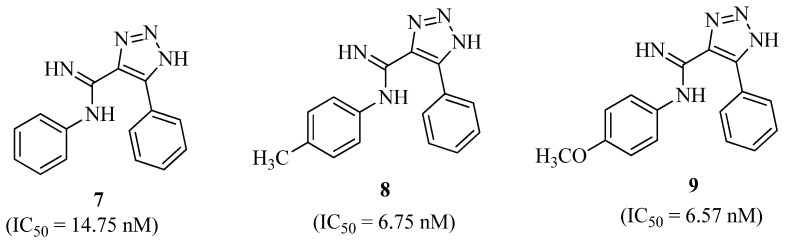
1,2,3-Triazole-4-carboximidamide derivatives.

A novel family of 1,2,3-Triazole-4-carboximidamide compounds was successfully developed for their DPP-IV inhibitory activity. When compared to the other drugs examined, compounds **7**, **8**, and **9** (Figure 5) displayed excellent inhibitory effects against DPP-IV, with IC_50_ values of 14.75, 6.75, and 6.57 nM, respectively. Compound **7**, at a dose of 10 mg/kg, enhanced glucose tolerance during OGTT in mice. Chronic treatment with compound **7** for 14 days in diabetic Wistar rats resulted in a significant drop in blood glucose levels, equivalent to the impact of Sitagliptin employed as a conventional treatment [32].

**Figure 6:**
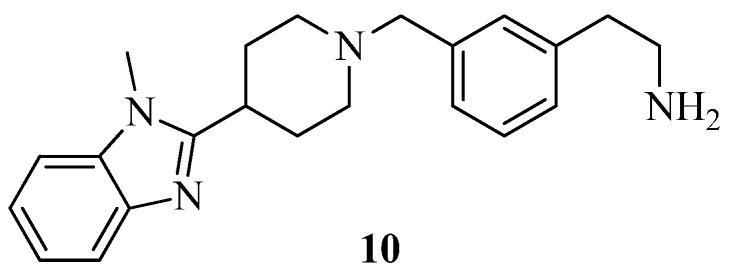
Benzimidazole derivative.

Six viable compounds were found using SBDD methods, and it was determined that compound **10** (Figure 6) had the highest docking score, i.e., −10.463 K.cal/mol with the DPP-IV enzyme (PDB ID-2ONC). Molecular dynamics (MD) simulations were also used to confirm the protein–ligand complex’s stability. Alogliptin and compound **10** were revealed to have a root-mean-square deviation (RMSD) smaller than 2.0 Å [33].

**Figure 7:**
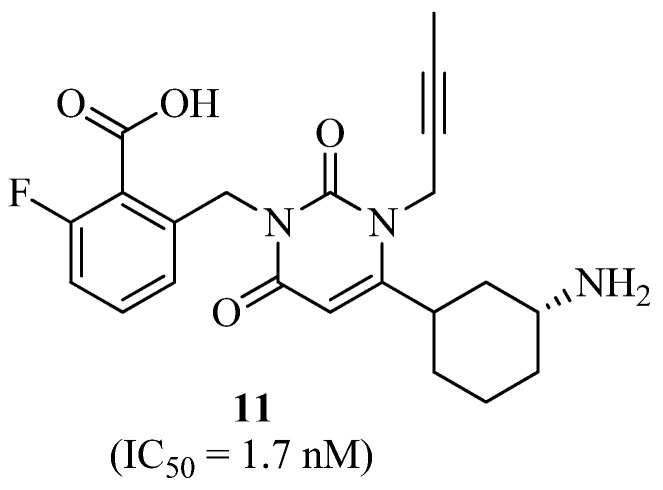
Uracil derivative containing benzoic acid as DPP-IV inhibitor.

To interact with Try629 and Lys554 at the S2′ position, novel uracil analogues with benzoic acid moieties at the N3 position were synthesized and tested for their DPP-IV inhibitory efficacy. From SAR studies, compound 11 was selected as the best candidate as a strong and selective DPP-IV inhibitor (IC_50_ = 1.7 nM). Based on docking data, it seems that additional salt bridging between the carboxylic acid group and the primary amine group of Lys554 plays a crucial role in increasing activity. According to the findings, compound **11** (Figure 7) showed no cytotoxicity in human hepatocyte L-O2 cells at doses up to 50 μM. Later, in vivo testing revealed that the ester of compound **11** considerably enhanced normal mice’s glucose tolerance. According to the study’s results, compound **11** has the potential to be a safe and effective medication for T2DM [34].

**Figure 8:**
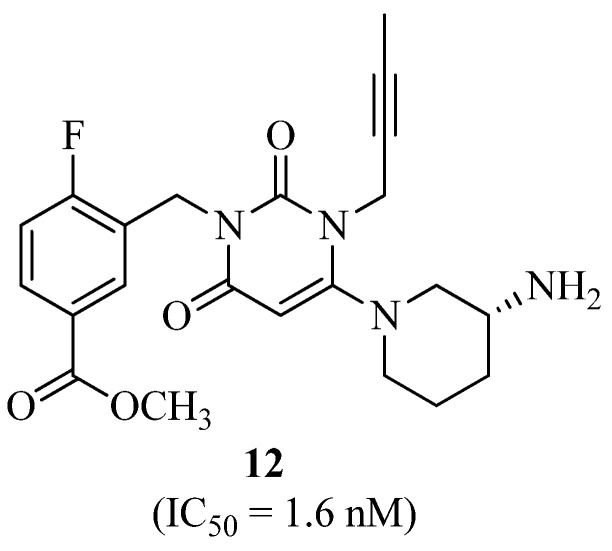
Aminopiperidin-dioxopyrimidin derivative as DPP-IV inhibitor.

Qing Li et al. [35] reported another DPP-IV inhibitor with minor modifications in compound **11** [**12**, methyl 3-((4-((*R*)-3-aminopiperidin-1-yl)-3-(but-2-ynyl), Figure 8] displaying excellent resultst in vivo.

From the above investigations, it was observed that oxygen and nitrogen heteroatoms play an important role in binding with receptors, resulting in significant conformational changes leading to potential biological activities. This led us to review the literature concerning the anti-diabetic activity of oxadiazoles.

## *3.* Oxadiazoles as Potential Nuclei 

**Figure 9:**
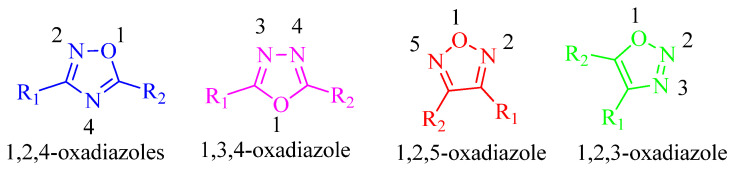
Several regioisomeric forms of oxadiazole rings.

**Figure 10:**
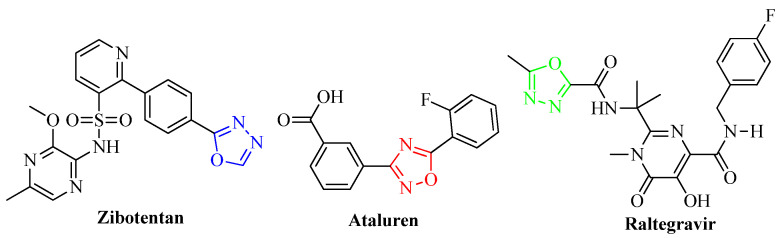
Oxadiazole structure-containing drugs.

Owing to their unique properties, oxadiazoles have attracted the attention of scientists in the disciplines of polymer and material science. These compounds feature five-membered rings comprised of two carbons, two nitrogens, and one oxygen atom (Figure 9). The number of patent applications for oxadiazoles has climbed to a total of 646 over the last nine years. This suggests that the scientific community has attached great importance to this class of chemicals [36]. Zibotentan, a potential anticancer agent, and ataluren (Figure 10), a potential therapeutic for cystic fibrosis, are two examples of oxadiazole-containing compounds that are now participating in late-stage clinical studies [37]. The only compound available on the market that currently contains oxadiazole is an antiviral drug called raltegravir (Figure 10), which is used to treat HIV infection [38]. Oxadiazoles are now being included in an increasing number of therapeutic approaches in a wide range of disease areas, including but not limited to diabetes [39], obesity [40], inflammation [41], cancer [42], and infection [43].

A wide array of applications have been found for oxadiazole rings in drug development projects. It has been shown that including them in the pharmacophore improves ligand binding in various ways. The carbonyl groups of molecules such as esters, amides, carbamates, and hydrogenated hydroxamic esters may be replaced with oxadiazoles, constituting another use for these types of molecules [44,45,46].

Oxadiazole rings exist in form of numerous regioisomers, including 1,2,4-, 1,3,4-, and 1,2,5-isomers (Figure 9). Because the side chains R1 and R2 of the 1,2,5-regioisomer are orientated in a different way compared to that of the side chains in the other two isomers, this isomer is found less often. Oxadiazoles may display different regioisomeric configurations, but they always contain the same R1 and R2 side chains. As a consequence, the positions of these side chains are quite similar. It is envisaged that matching pairings would have identical overall molecular structures and, as a result, would form bonds in the same way. The exceptional hydrogen bond acceptor properties of oxadiazoles and the distinct hydrocarbon bonding potentials of their regioisomers have been established [47,48,49]. We will now investigate how oxadiazoles may help in treating diabetes.

## 4. An Overview of the Potential Use of Oxadiazole Derivatives as Anti-Diabetic Drugs 

1,3,4-Oxadiazole and 1,2,4-oxadiazole have the potential to be used for many therapeutic applications [50,51,52,53,54,55,56].

Omarigliptin is a sulfonamide-containing moiety that inhibits the DPP-IV enzyme to achieve its antihyperglycemic activity [57]. Investigations into its pharmacokinetic properties have shown that it may be given on a once-weekly basis, which distinguishes it from all other DPP-IV inhibitors. The derivatives of 2-cyanopyrrolidine are classified as glycine-based inhibitors, which fall within the larger category of peptidomimetic inhibitors [58,59]. The presence of a nitrile group on the five-membered pyrrolidine ring in 2-cyanopyrrolidine derivatives is indicative of their capacity to exert reversible nanomolar inhibition of the DPP-IV enzyme [60]. We reasoned that compounds based on oxadiazoles would be able to provide an alternative therapy for diabetes for not only controlling glucose levels but also preventing the progression of atherosclerosis and other complications associated with diabetes.

**Figure 11:**
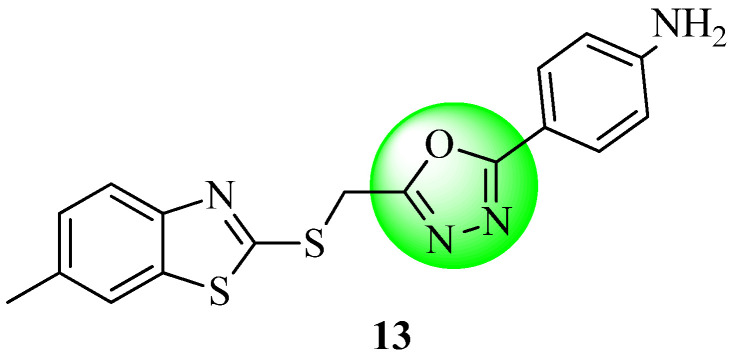
Benzothiazole tethered with 1,3 4-oxadiazole derivatives as an anti-diabetic oxadiazole derivative.

Kumar et al. [61] discovered that some 2-((benzothiazol-2-ylthio) methyl)-5-phenyl-1,3,4-oxadiazole derivatives have the potential to act as anti-diabetic compounds. Using glibenclamide as the reference, each of the synthetic compounds were evaluated for their capacity to combat diabetes in an animal model. At a dose of 350 mg/kg p.o., compound **13** (Figure 11) displayed the most noticeable effect of all molecules, even though all showed a significant activity (orally).

**Figure 12:**
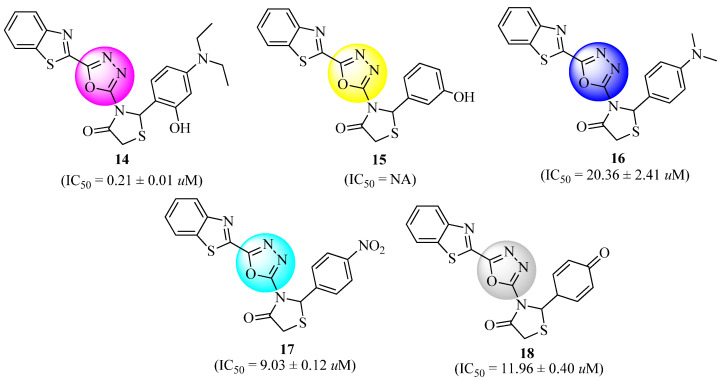
Synthetic analogues of benzothiazole-1,3,4-Oxadiazole-4-thiazolidinone.

Benzothiazole-1,3,4-oxadiazole-4-thiazolidinone hybrid derivatives were synthesized by Bhutani et al. [62]. OGTT in non-diabetic rats and streptozotocin-induced diabetic rat models were used to evaluate seven compounds with the highest docking scores. The blood glucose levels were significantly reduced with all of the studied compounds, ranging from good to moderate. The anti-diabetic effects of the three compounds (**14**, **15** and **16**, Figure 12) were superior to those of the conventional medicine pioglitazone, showing a glucose concentration of 178.32 ± 1.88 mg/dL, compared to the lower concentrations of glucose of 157.15 ± 1.79 mg/dL, 154.39 ± 1.71 mg/dL, and 167.36 ± 2.45 mg/dL reported for **14**–**16**. Acarbose (IC_50_ = 18.5 ± 0.20 µM) was found to be the most potent inhibitor of alpha-glucosidase among the seven derivatives examined. However, three of its derivatives, compounds **14**, **17**, and **18** (Figure 12), displayed lower IC_50_ values (0.21 ± 0.01 µM, 9.03 ± 0.12 µM and 11.96 ± 0.40 µM, respectively), suggesting that they were less effective than the ordinary acarbose. In other words, these innovative hybrids could be used as a basis for the development of novel agents [62].

**Figure 13:**
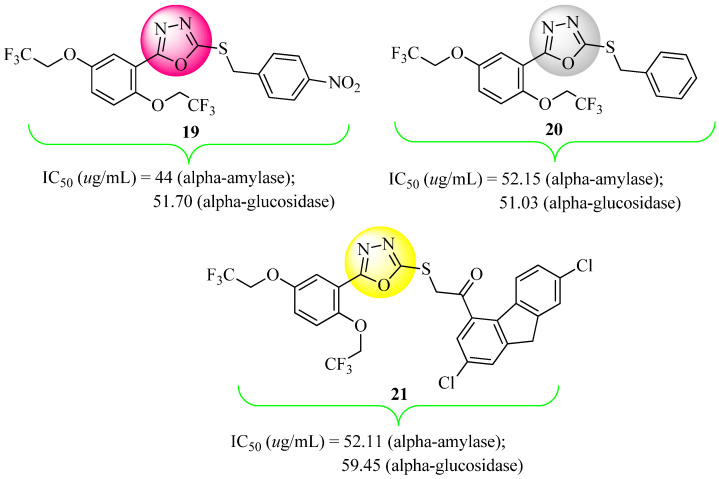
1,3,4-Oxadiazole-2-thiol derivatives.

New 5-(2,2,2-trifluoroethoxy)phenyl-1,3,4-oxadiazol-2-thiol derivatives have been synthesized and analyzed for their biological activities in vitro and in vivo. In comparison to acarbose (IC_50_ = 34.71 μg/mL), these compounds showed α-amylase inhibitory activities in the IC_50_ range of 40.00–80.00 μg/mL. Compounds **19** and **20** were the ones that exhibited the highest levels of activity in vitro compared to the other synthesized compounds. Animal experiments showed that compounds **19**, **20**, and **21** (Figure 13) were able to reduce glucose levels in Drosophila, but displayed a 17–30% lower capacity than acarbose. It was shown that compounds **19** and **20** exhibited the highest activity among the produced compounds. In this study, compounds **19**, **20**, and **21** were revealed to be good candidates for further research as possible anti-diabetes drugs [63].

**Figure 14:**
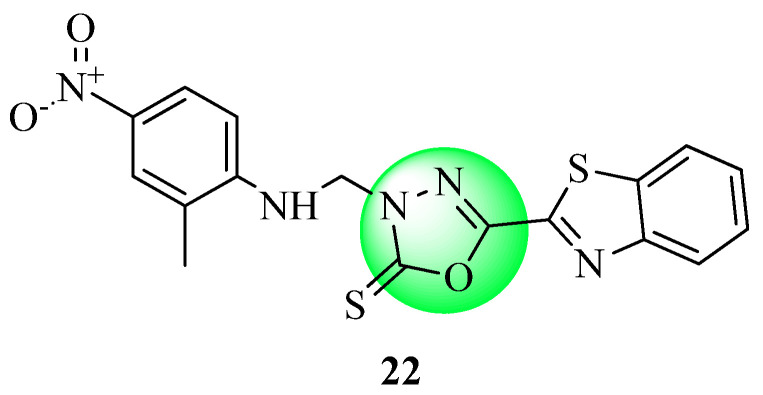
Benzothiazole clubbed oxadiazole-Mannich base.

New benzothiazoles clubbed oxadiazole-Mannich bases (M-1 to M-22) synthesized by Bhutani et al. were evaluated using OGTT and STZ-induced diabetes in normal rats. Glucose levels were reduced with compound M-14 (**22**, Figure 14) (161.39 ± 4.38 mg/dL) in the STZ model, which was equivalent to treatment with glibenclamide (140.29 ± 1.24 mg/dL). The antihyperglycemic efficacy of the other substances tested ranged from fair to excellent [64].

**Figure 15:**
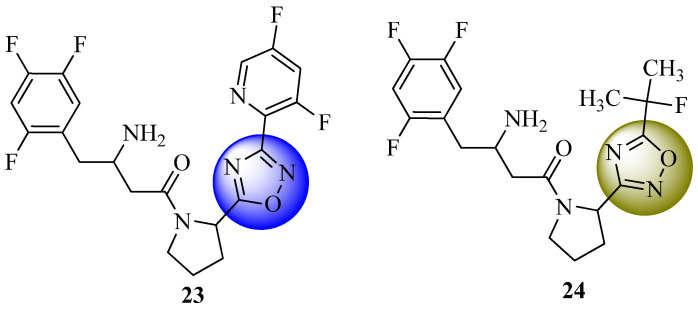
β-homophenylalanine-based inhibitors of DPP-IV.

Nordhoff et al. [65] demonstrated that amide substitutions enhanced the absorption, distribution, metabolism, and excretion (ADME) characteristics of a series of β-homophenylalanine-based inhibitors of DPP-IV. Thanks to the efforts of this group, a new class of powerful and selective DPP-IV inhibitors with an appealing pharmacokinetic profile and good performance was synthesized and evaluated in an animal model of diabetes. Compounds **23** and **24** (Figure 15) from this new series of compounds were shown to display interesting pharmacokinetic properties and to work very well in animal models of diabetes.

**Figure 16:**
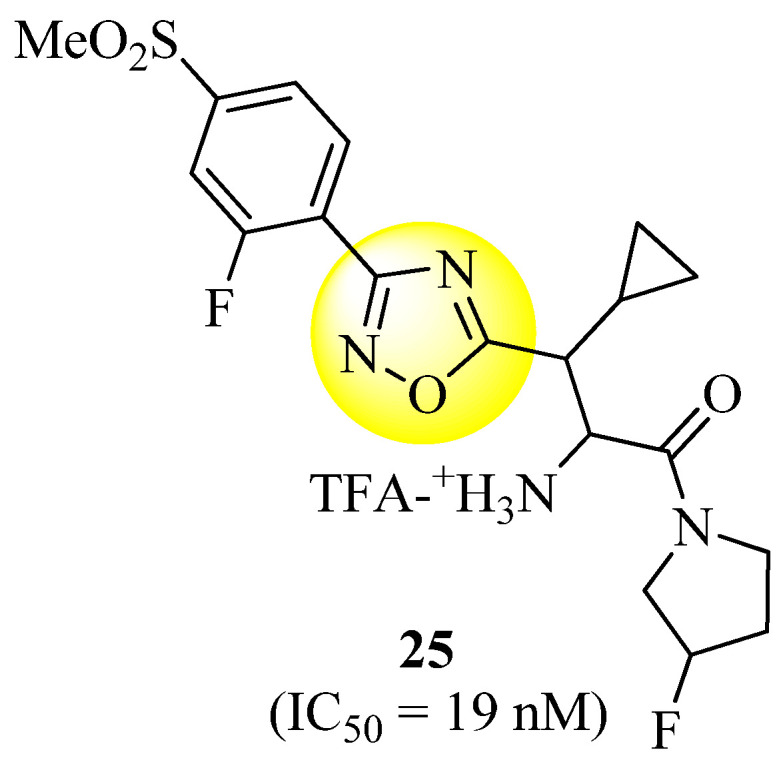
1,2,4-Oxadiazole derivative as a potent DPP-IV inhibitor.

A new class of powerful, selective, and orally accessible DPP-IV inhibitors has been discovered by Xu et al. [66] Without an electrophilic trap, they are among the most powerful chemicals known to date. Regarding DPP-IV homologs, compound **25** (Figure 16) was shown to have a higher selectivity. However, their short half-life, observed after oral treatment in rats and dogs, led to further research being stopped on these compounds.

**Figure 17:**
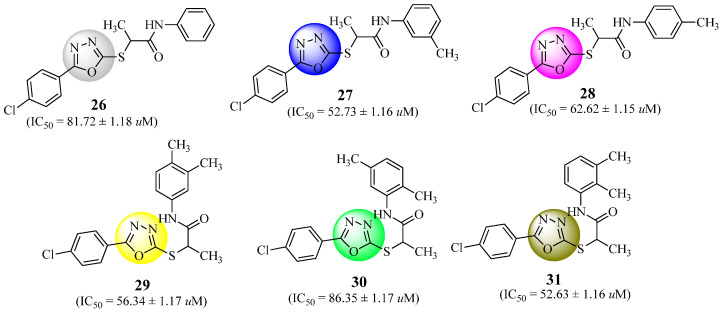
Oxadiazole derivatives with strong α-glycosidase inhibitory activity.

*N*-aryl/aralkyl derivatives of 2-methyl-2-{5-(4-chlorophenyl)-1,3,4-oxadiazole-2-ylthiol}acetamides were synthesized and the α-glucosidase inhibitory potential of each drug was examined. Compounds **26**–**31** (Figure 17) displayed a strong α-glycosidase inhibitory activity (IC_50_ of 81.72 ± 1.18, 52.73 ± 1.16, 62.62 ± 1.15, 56.34 ± 1.17, 86.35 ± 1.17, 52.63 ± 1.16 µM, respectively). These results were validated by molecular modelling and ADME predictions. It was then possible to synthesize a library of compounds using ordinary basic materials possibly leading to the development of new medicines [67].

**Figure 18:**
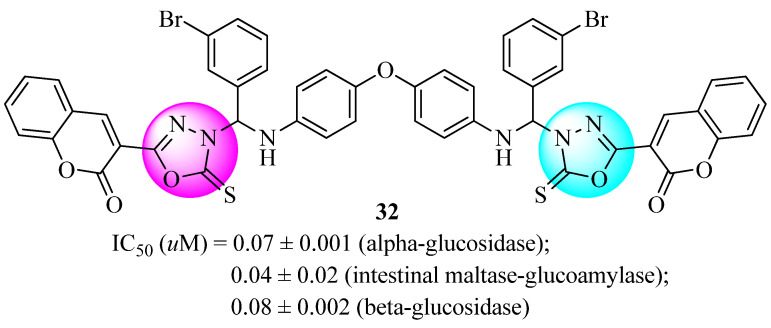
Anti-diabetic drugs derived from diamine-bridged coumarinyl oxadiazoles.

One-pot multicomponent design and synthesis of three series of diamine-bridged *bis*-coumarinyl oxadiazole conjugates were reported by Kazmi et al. [68]. The conjugates produced were tested for their ability to inhibit glucosidases. With an IC_50_ value of only 0.07 ± 0.001 μM (acarbose: 38.2 ± 0.12 μM), compound **32** (Figure 18), including the 4,4′-oxydianiline linker, was shown to be the primary and selective inhibitor of alpha-glucosidase enzymes. Its inhibitory activity was about 545 times higher compared to conventional drugs. Compound **32** was also shown to be a strong inhibitor of intestinal maltase-glucoamylase (IC_50_ = 0.04 ± 0.02 μM) compared to acarbose (IC_50_ = 0.06 ± 0.01 μM). With an IC_50_ value of 0.08 ± 0.002 μM, this compound was reported to be the primary inhibitor of the β-glucosidase enzyme. The mechanism of the inhibition was investigated using Michaelis–Menten kinetic studies. All the generated molecules were docked against the glucosidase enzyme. According to the obtained results, numerous interactions were observed with catalytic residues in a coordinated manner, which might stabilize inhibitors in the active site. In addition, β-glucosidase inhibitors were successfully identified via the use of molecules having strong binding interactions with amino acid residues.

**Figure 19:**
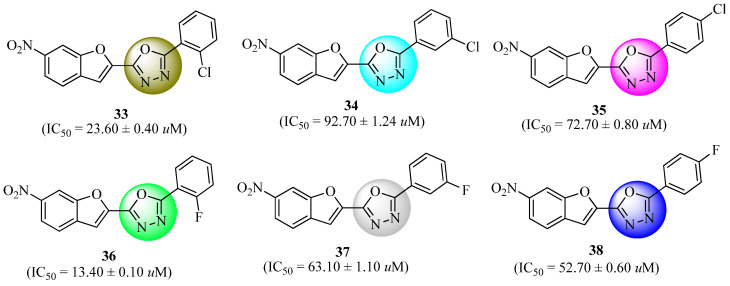
Nitrobenzofuran-1,3,4-Oxadiazoles derivatives.

Taha et al. [69] designed and synthesized twenty 5-aryl-2-(6′-nitrobenzofuran-2′-yl)-1,3,4-oxadiazole derivatives and tested them for their ability to inhibit α-glucosidase. Compared to acarbose (IC_50_ = 856.45 ± 5.60μM), compounds with hydroxyl groups and halogens (compounds **33**–**38**, Figure 19) were shown to be five to seventy times more active, with IC_50_ values in the range of 12.75 ± 0.10 to 162.05 ± 1.65 μM. A hybrid family of oxadiazole and benzofuran compounds is now being studied for its ability to block α-glucosidase. Researchers may be prompted to use these results for the treatment of hyperglycemia. Within docking studies, hydrogen bonds and arene–arene interactions were shown to be the primary means of interactions with Glu 276, Asp 214, and Phe 177.

Anti-diabetic drugs derived from 1,3,4-oxadiazoles have been discovered by Ullah et al. [70]. The ability of these newly synthesized compounds to inhibit α-glucosidase activity was investigated. As compared to acarbose (38.45 ± 0.80 μM), all compounds showed strong inhibitory activities with IC_50_ values ranging from 0.80 ± 0.1 to 45.1 ± 1.7 μM. Thirteen compounds revealed potential inhibitory actions, though only one molecule (IC_50_ = 45.1 ± 1.7 μM) was found to be less active. Among all the synthesized derivatives, one compound (IC_50_ = 0.80 ± 0.1 μM) demonstrated the most promising inhibitory efficacy.

**Figure 20:**
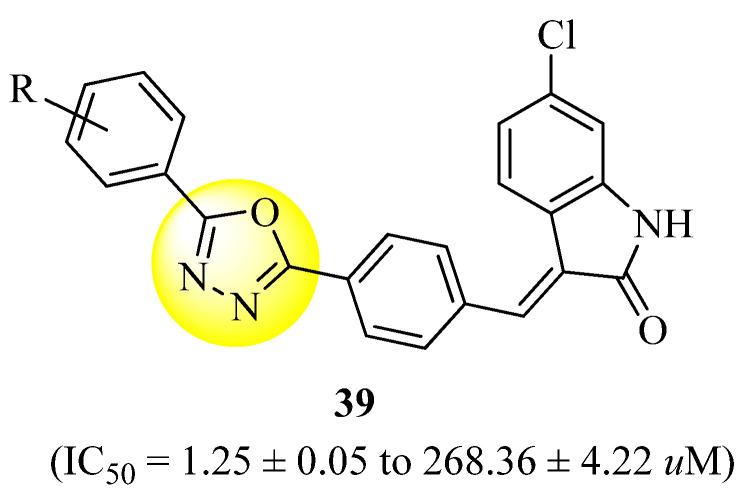
α-Glucosidase inhibitors based on oxadiazole hybrids.

Taha et al. [71] discovered hybrid analogues of oxindole-based oxadiazoles (based on the structure of compound **39**, Figure 20) as potential α-glucosidase inhibitors. As compared to acarbose (IC_50_ = 895.09 ± 2.04 µM), all compounds were shown to be powerful inhibitors of this enzyme, with IC_50_ values ranging from 1.25 ± 0.05 to 268.36 ± 4.22 µM. In this work, a new series of powerful α-glucosidase inhibitors have been discovered, suggesting further investigations.

**Figure 21:**
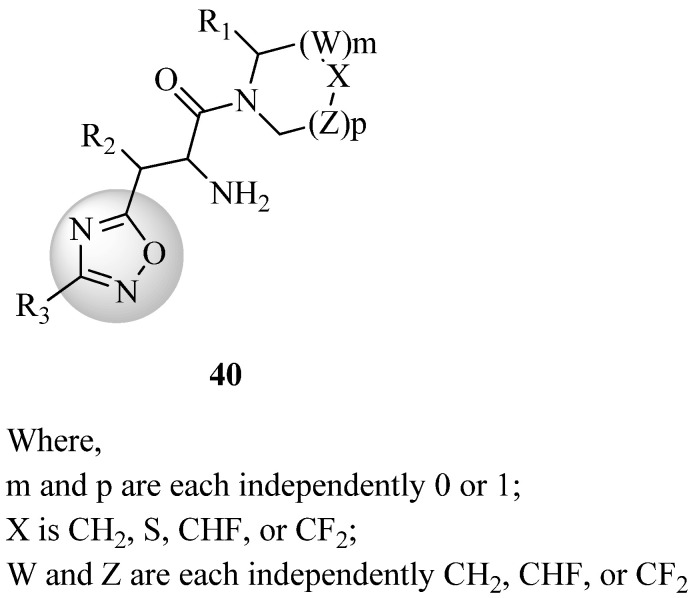
DPP-IV inhibitors derived from 1,2,4-oxadiazole.

New 1,2,4-oxadiazole derivatives, whose structures were reported in the US patent issued by Xu et al. [72] (compound **40**, Figure 21), are DPP-IV enzyme inhibitors and may be used to treat or prevent disorders involving this enzyme, such as diabetes, and more specifically type 2 diabetes. A pharmaceutical composition including these compounds and their application in the prevention or treatment of illnesses involving the DPP-IV enzyme are also contemplated by this invention.

**Figure 22:**
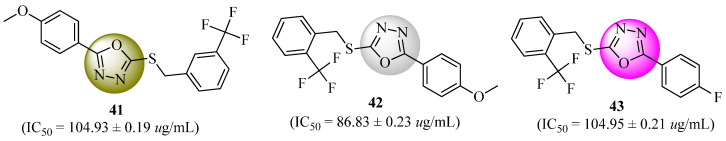
Inhibitors of alfa-amylase activity based on 1,3,4-oxadiazole derivatives.

Hamdani et al. [73] synthesized three 1,3,4-oxadiazole derivatives (compounds **41**, **42**, and **43**, Figure 22) and used X-ray diffraction, density functional theory (DFT), and other methods to demonstrate their inhibitory potential of α-amylase. X-ray diffraction and other spectro-analytical methods were employed to validate the structures of the obtained compounds, which were prepared in excellent yields (70–83%). In addition to validating X-ray data, DFT calculations also examined charge dispersion and reactivity, utilizing frontier molecular orbitals and molecular electrostatic potential (MEP) approaches. α-amylase inhibition experiments were used to determine the enzymatic inhibitory capacity of the produced compounds (**41**, **42**, and **43**). Compound **42** displayed a low IC_50_ value of 86.83 ± 0.23 μg/mL, which indicates its strong ability to inhibit α-amylase.

The anti-diabetic properties of oxadiazole derivatives were studied in silico using the DFT approach, employing the B3LYP version with compounds set by Ibrahim et al. [74]. Four models were generated using the Genetic Function Algorithm (GFA). Based on the obtained results, researchers determined that Model 1 had the highest LOF (0.030552), R2 (0.09681), R2adj (0.09567), Q2CV (0.09364), and R2 (0.06969) values. Findings from molecular docking indicated that few ligands had the greatest docking scores of −9.9 kcal/mol among the co-ligands. The docking ratings produced here were shown to be in concordance with findings from previous studies. As a result of this work, anti-diabetic drugs with improved inhibitory action against α-glucosidase could potentially be developed.

**Figure 23:**
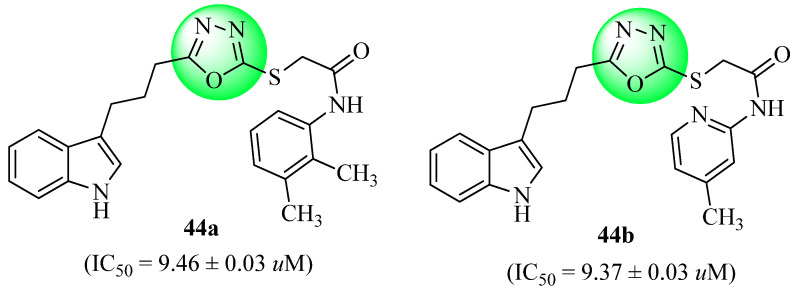
Derivatives of the 1,3,4-oxadiazole-2-thiol compound.

In order to investigate the sequential conversion of indolyl butanoic acid into 1,3,4-oxadiazole-2-thiols, Nazir et al. [75] employed several chemical transformations. Several different amine derivatives were reacted with 2-bromoacetyl bromide to serve as an electrophile, leading to the production of 2-bromo-*N*-phenyl/arylacetamides in a series of operations that ran in parallel with one another. A nucleophilic 1,3,4-oxadiazole-2-thiol analogue was then applied to the electrophilic compounds to produce a variety of N-substituted derivatives (compounds **44a** and **44b**, Figure 23). In this study, the anti-diabetic potential of all produced compounds was first examined through the inhibition of the α-glucosidase enzyme, and then by studying them in silico. In addition, their hemolytic activity was used to determine their cytotoxicity profile, and all of the compounds were shown to display minimal cytotoxicity. The most active compounds (**44a** and **44b**) had IC_50_ values of 9.46 ± 0.03 µM and 9.37 ± 0.03 µM, respectively. As a result, they may serve in future studies to develop more efficient anti-diabetic drugs, as they showed excellent to moderate inhibitory potentials (IC_50_ = 12.68 ± 0.04 to 37.82 ± 0.07 µM, respectively).

## 5. Structural Activity Relationship (SAR) 

**Figure 24:**
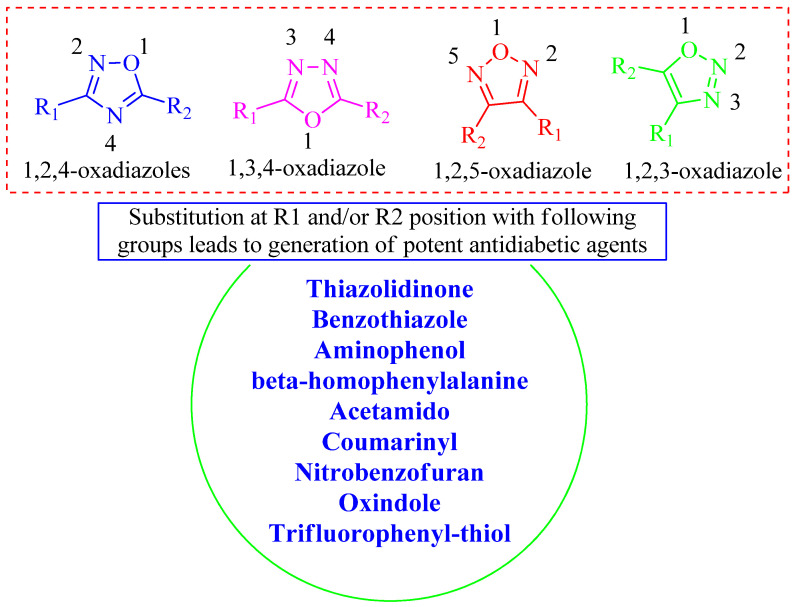
Predicted SAR of oxadiazoles.

A brief structural activity relationship (SAR) is depicted in Figure 24. It was observed that almost all the potent derivatives were found to hold substitutions with different heterocyclic compounds or alkyl groups. Substitution with benzothiazoles and thiazolidinones leads to potent in vivo activity and displays significant overall anti-diabetic activities. The transformation of oxadiazole into the acetamido functional group has led to the development of potential anti-diabetic agents, which were revealed as excellent DPP-IV inhibitors. However, many more substitutions, such as aminophenyl, β-homophenylalanine, coumarinyl, nitrobenzofuran, oxindole, trifluorophenyl-thiol, etc., could act as potential anti-diabetic agents, and especially as potential DPP-IV inhibitors.

## 6. Conclusion

1,3,4-Oxadiazole and 1,2,4-oxadiazole derivatives can be used in a variety of ways within the medical sector. Oxadiazoles possess diverse pharmacological activities, including anti-diabetic potential, and specifically acting as DPP-IV inhibitors. It has been reported that oxadiazoles fused with benzothiazoles, 5-(2,2,2-trifluoroethoxy)phenyl, β-homophenylalanine, 2-methyl-2-{5-(4-chlorophenyl), diamine-bridged *bis*-coumarinyl, 5-aryl-2-(6′-nitrobenzofuran-2′-yl), nitrobenzofuran, and/or oxindoles demonstrate potential anti-diabetic activities. Furthermore, the most potent derivatives were obtained by substituting sulfur-containing heterocyclic compounds, as the sulfur atom has the potential to form interactions with the target enzymes. It was observed that almost all the potent derivatives were found to possess substitutions with different heterocyclic compounds or alkyl groups. Many of these derivatives induced strong conformational changes to the DPP-IV enzyme, resulting in excellent anti-diabetic activity. From this analysis, we came to the conclusion that oxadiazoles have properties that make them potent drugs. Further research is needed to consider them as valuable drugs to treat diabetes, especially as DPP-IV inhibitors. As discussed above, substitutions at the R1 and R2 positions leads to more potent and effective DPP-IV inhibitors. It may be possible to design some potent derivatives in future by appending different heterocyclic and alkyl groups at the R1 and R2 positions. It is a great challenge to maintain the binding mode of the derivatives following substitutions. After substitution, each derivative can be screened through molecular docking studies to determine its ability to cause conformational changes in the DPP-IV enzyme. 

## Data Availability

Not applicable.
